# Miller-Fisher Syndrome Following Influenza A Infection

**DOI:** 10.7759/cureus.56064

**Published:** 2024-03-12

**Authors:** Shiho Mitsuhashi, Asuka Suzuki, Koji Hayashi, Mamiko Sato, Yuka Nakaya, Naoko Takaku, Yasutaka Kobayashi

**Affiliations:** 1 Department of Rehabilitation Medicine, Fukui General Hospital, Fukui, JPN; 2 Graduate School of Health Science, Fukui Health Science University, Fukui, JPN

**Keywords:** methyl prednisolone, intravenous immunoglobulins (ivig), miller fisher syndrome (mfs), guillain-barre syndrome (gbs), influenza virus type a

## Abstract

Miller-Fisher syndrome (MFS), characterized by ophthalmoplegia, ataxia, and areflexia, is a Guillain-Barré syndrome (GBS) variant. It is well-known that the causative antibody for MFS is anti-GQ1b antibody. This report describes a rare case of MFS with not only anti-GQ1b antibodies but also anti-GT1a antibodies following Influenza A infection. The patient, a 47-year-old woman, contracted Influenza A three weeks before admission. She complained of double vision followed by areflexia, ataxia in the four extremities, and complete gaze palsy. She was treated with intravenous methylprednisolone pulse and intravenous immunoglobulin therapies. Her neurological symptoms were recovered after these immunotherapies.

## Introduction

Miller-Fisher syndrome (MFS), characterized by ophthalmoplegia, ataxia, and areflexia, is a Guillain-Barré syndrome (GBS) variant associated with anti-GQ1b IgG antibody [1]. In both MFS and GBS, it has been suggested that there is a relationship with antecedent infections, including respiratory tract infection or gastrointestinal illness [2]. Regarding causative microorganisms, Campylobacter jejuni and cytomegalovirus are the most commonly identified [2]. It is reported that whereas other possible infectious causes include Epstein-Barr virus, Mycoplasma pneumoniae, and Haemophilus influenzae, Influenza A infection is a relatively rare cause of GBS or MFS [2,3]. Although the detailed mechanism is unknown, GBS or MFS associated with influenza infection rarely test positive for ganglioside antibodies [2]. Herein, we describe a rare case of MFS following Influenza A infection with anti-GQ1b and anti-GT1a antibodies.

## Case presentation

**Figure 1:**
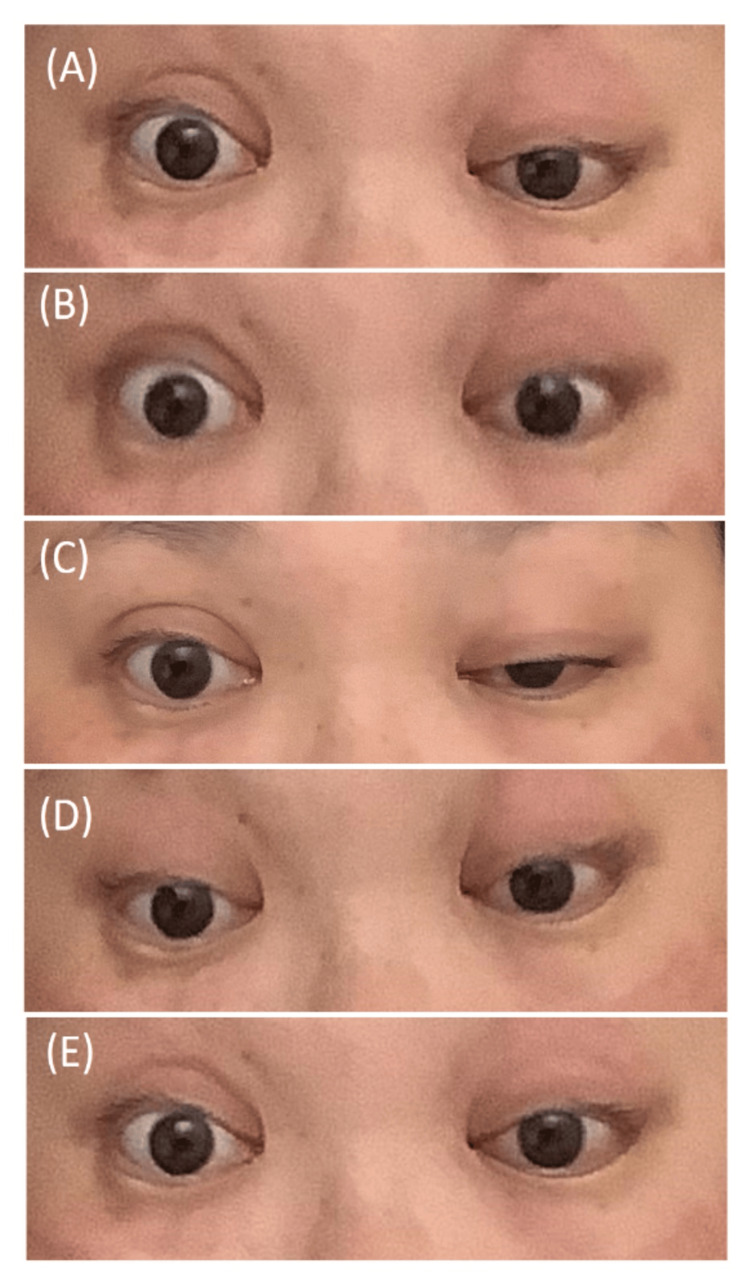
The clinical pictures of eye movements on day 4. As the left blepharoptosis was particularly noticeable in the front view, the gaze was evaluated with the eyelids held down. Her eyes remained fixed in the midline, no matter which direction she tried to be turned: (A) right-side gaze, (B) top-center gaze, (C) center gaze, (D) bottom-center gaze, and (E) left-side gaze.

A 47-year-old woman became infected with Influenza A, confirmed by a rapid antigen test, three weeks before admission. She had a fever followed by upper respiratory tract symptoms. At this time, she had a fever for three days. After the fever subsided, a persistent cough continued for two weeks. Other symptoms, including diarrhea, were not noted. On the morning of admission, she noticed a double vision and visited our hospital. Vital signs were unremarkable except for mild high blood pressure (144/105 mmHg). Neurological examination revealed upward rotation restriction of the left eye and diplopia in front and upward views. No other neurological symptom was noted, including decreased deep tendon reflex or ataxia. Blood tests were unremarkable except for mildly elevated aspartate aminotransferase, alanine aminotransferase, and gamma-glutamyl transpeptidase. Cerebrospinal fluid (CSF) tests revealed normal results, including protein level (Table 1). Nerve conduction studies in both motor and sensory nerves, including median, ulnar, tibial, and peroneal nerve, were all normal. Brain MRI was unremarkable. We suspected paralysis of the left superior rectus muscle and chose intravenous methylprednisolone pulse therapy (IVMP; 1,000 mg/day, three days). On day 2, she developed bilateral abduction restriction, mild ataxia, and areflexia in extremities. On day 4, complete gaze palsy was developed and blepharoptosis was noted (Figure 1).

**Figure 2:**
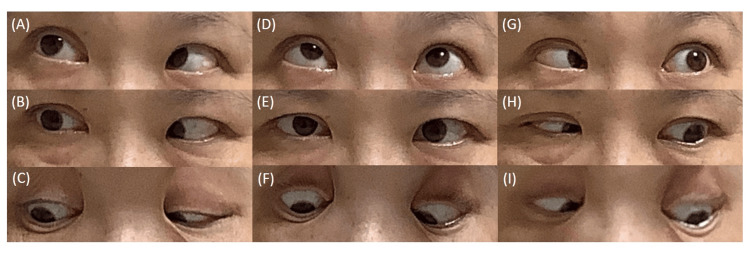
The clinical pictures of eye movements on day 55. Although the eye movement disorder during lateral gaze remained slightly, it had significantly improved. Additionally, blepharoptosis was nearly completely recovered: (A) top right-side gaze, (B) right-side gaze, (C) bottom right-side gaze, (D) top center gaze, (E) center gaze, (F) bottom-center gaze, (G) top left-side gaze, (H) left-side gaze, and (I) bottom left-side gaze.

On day 5, CSF tests revealed mononuclear cell-dominated pleocytosis but normal protein (Table 1). Based on the clinical findings, we suspected MFS and started intravenous immunoglobulin (IVIg) therapy on days 4 to 6. On day 12, a restudy of CSF revealed proteinocytological dissociation (Table 1). Although her symptoms were poorly improved, IVMP was added between days 14 and 16. In addition, it is disclosed that anti-GQ1b immunoglobulin G (IgG) antibody and anti-GT1a IgG antibody were positive on day 17. Based on these findings, we diagnosed her with MFS. On day 19, areflexia and gaze palsy were slightly improved. Although gaze palsy and diplopia were improved gradually but preserved, IVMP was administered again on days 38 to 40. On day 55, her gaze palsy and ptosis showed significant improvement, but diplopia during side gaze persisted (Figure 2). Neurological examination revealed that the ataxia had completely resolved and deep tendon reflexes in four extremities were nearly completely normalized. She was discharged from our hospital on day 57.

**Table 1:** The result of cerebrospinal fluid tests. IgG, immunoglobulin G

Inspection items	On admission	On day 5	On day 12	Reference range
Cell count	1/µL	10/µL	3/µL	<5
Mononuclear cell	100%	83.9%	90.0%	
Polynuclear cell	0%	16.1%	10.0%	
Protein	30.0 mg/dL	30.1 mg/dL	51.7 mg/dL	<40
Glucose	61 mg/dL	102 mg/dL	75 mg/dL	50-75
IgG index	0.54	0.63	0.55	0.7-0.8

## Discussion

We describe a rare case of MFS triggered by Influenza A infection. The patient became infected with Influenza A infection three weeks before admission. Neurological findings were typical for MFS, featuring a triad of complete gaze palsy, ataxia, and areflexia, while these symptoms gradually manifested. In addition, CSF findings were normal on admission, but proteinocytological dissociation was noted on day 12. Additionally, it was disclosed that anti-GQ1b IgG and anti-GT1a IgG antibodies were positive. No paralysis or sensory disturbance was noted. Moreover, the patient’s neurological symptoms were recovered through immunotherapy, including IVIg and IVMP. Based on these findings, we diagnosed the patient with MFS following Influenza A infection.

A few case reports about MFS or GBS related to influenza infection exist. The major pathogen of MFS or GBS is C. jejuni [1], but the features of influenza are quite different from C. jejuni. The influenza cases are characteristic of individuals younger than 65, and none had antiganglioside antibodies [2]. Indeed, Afflu et al. reported a 59-year-old male case of MFS following Influenza A infection [3]. This report described that although the ganglioside antibody was negative, the patient was diagnosed with MFS by a triad of MFS and CSF findings, including albuminocytologic dissociation. In addition, Costiniuk et al. reported a 17-month-old toddler case of MFS following Influenza A (pH1N1) infection [4]. This case did not perform the test for anti-GQ1b antibody. The patient was diagnosed with MFS by neurological examination, including blepharoptosis, areflexia, and ataxic gait. On the other hand, as far as we know, only one case has been reported about MFS related to Influenza A infection with the ganglioside antibody [5]. In this report, a 36-year-old male case tested positive for anti-GQ1b IgG and anti-GT1a IgG antibodies. These ganglioside antibodies detected were the same as in our case.

The ganglioside GQ1b is abundant in the paranodal region of the extramedullary portion of the oculomotor, trochlear, and abducens nerves, a subpopulation of large neurons in dorsal root ganglia, and muscle spindles in limbs [6-8]. Therefore, the anti-GQ1b antibody is a main causative pathogen for MFS [6]. On the other hand, the ganglioside GT1a is abundant in glossopharyngeal and vagal nerves [9]. In GBS cases, patients with anti-GT1a IgG often had cranial nerve palsy (ophthalmoparesis, 57%; facial palsy, 57%; bulbar palsy, 70%), and 39% needed artificial ventilation [9]. It has been reported that double positive cases for anti-GQ1b and anti-GT1a antibodies developed pure MFS [5] or symptoms of overlapping MFS/GBS [10]. While symptoms can vary in cases double positive for anti-GQ1b and anti-GT1a antibodies, our case presented solely with symptoms consistent with MFS.

Regarding the treatment, we employed not only IVIg, recognized as one of the gold standards for MFS or GBS, but also IVMP in our case. There are some reports about the successful treatment of MFS by IVMP following IVIg [11-12]. Here, we chose IVMP as the initial treatment in the phase when the diagnosis for MFS was not yet confirmed. Subsequently, we used IVIg as a standard treatment, followed by adding IVMP to shorten the disease duration. Whereas the effectiveness of IVMP for MFS must be judged carefully, our patient did not experience any side effects and was doing well.

## Conclusions

We presented a rare case of MFS following Influenza A infection, supported by evidence of ganglioside antibodies, including anti-GQ1b and GT1a antibodies. In some cases with MFS, the appearance of symptoms is not simultaneous, which makes diagnosis difficult in the initial stages. Although the phenotype may differ depending on the type of ganglioside antibodies tested positive, MFS was developed by two types of ganglioside antibodies, including anti-GT1a antibody in addition to anti-GQ1b antibody in our case. Additionally, we achieved successful treatment of MFS using not only IVIg but also IVMP. The additional use of IVMP may be considered as an option for MFS when recovery is delayed. More cases need to be accumulated to unveil the underlying mechanisms associated with MFS and influenza infection.
